# An enhanced teaching interface for a robot using DMP and GMR

**DOI:** 10.1007/s41315-018-0046-x

**Published:** 2018-03-08

**Authors:** Chunxu Li, Chenguang Yang, Zhaojie Ju, Andy S. K. Annamalai

**Affiliations:** 1 Centre for Computational Engineering, Swansea University, Swansea, SA1 8EN, UK; 2 School of Computing, University of Portsmouth, Portsmouth, PO1 3HE, UK; 3 Moray College, University of Highlands and Islands, Inverness, UK

**Keywords:** Teaching interface, Teleoperation, Dynamic movement primitive (DMP), Gaussian mixture regression (GMR), Dynamic time warping (DTW)

## Abstract

This paper develops an enhanced teaching interface tested on both a Baxter robot and a KUKA iiwa robot. Movements are collected from a human demonstrator by using a Kinect v2 sensor, and then the data is sent to a remote PC for the teleoperation with Baxter. Meanwhile, data is saved locally for the playback process of the Baxter. The dynamic movement primitive (DMP) is used to model and generalize the movements. In order to learn from multiple demonstrations accurately, dynamic time warping (DTW), is used to pretreat the data recorded by the robot platform and Gaussian mixture model (GMM), aiming to generate multiple patterns after the teaching process, are employed for the calculation of the DMP. Then the Gaussian mixture regression (GMR) algorithm is applied to generate a synthesized trajectory with smaller position errors in 3D space. This proposed approach is tested by performing two tasks on a KUKA iiwa and a Baxter robot.

## Introduction

With the advance of new robotic technology, the application of robots in both industry and social service fields has been widely used. The natural and friendly human–robot interaction plays an increasingly important role to bring robot technology into human daily life (Chin and Yue 2011). Especially, robotic skill learning receives much attention in the robotics area. Wherein, through imitation and skill learning from human operators, the robots are able to optimize the execution of a specific task. The research focusing on understanding the human motion based on teaching by demonstration (TbD) has attracted wide interest during the past decades (Li et al. 2017). In Li et al. (2014), a slave/master controller based TbD is developed to retain the kinematic constraint by using the tracking system. A trajectory learning approach for multi-robot interaction for welding task has been developed in Chernova and Veloso (2008). The ability of human skills transferred from demonstrators has a huge influence on robot intelligence, and is an important way of robot learning, successfully avoiding the difficulties of the control of the complex movement imitated from human.

The teaching process from a human operator transfers the motor skills to the imitator (robot) through recording the motion of the learning movement and generalized output Calinon et al. (2014). In Chin and Yue (2011), a research team develops a problem-based learning (PBL) method on an autonomous vacuum robot with mechatronics systems, by doing this, the students are able to have hands-on experience. In addition, an actuated dynamics technology based proportion-integral-derivative (PID) controller is proposed in Chin et al. (2016), and the global exponentially boundedness of the unactuated dynamics has been established. The acquisition of teaching data is realized through body sensation, which conveys human–computer dialogue effectively. This has a wide usage in intelligent identification and control system as a natural way of human–computer interaction. Common somatosensory devices include wear systems, such as 3DSuit, data gloves, and optical movement capture system, such as Microsoft’s Kinect somatosensory camera (Frick and Alberts 2006).

Gaussian mixture model (GMM) is a commonly used clustering algorithm. It approximates the complex distribution accurately. It first extracts a feature element of each unit in the sample, then uses the Gaussian mixture model to cluster these features as an object, finally obtaining the segmentation result. In Huang et al. (2005), the authors used continuous myoelectric signals and employed GMMs for multiple limb motion classification. In addition, earlier in the 19th century, scientists used a finite GMM whose parameters are estimated through the EM algorithm by estimating a probability density function of human skin color (Yang and Ahuja 1998). Playback process of a robot includes movement control and movement trajectory reproduction. Trajectory reproduction is employed when the encoded data is sent to regression techniques, such as Gaussian Mixed Regression (GMR) (Grollman and Jenkins 2008). Movement control is the result of mapping robot movement trajectories, which is aiming to acting the reproduction. To be precise, it is the playback process after the learning process from a demonstrator. In addition, human skill transfer can be achieved optimally by programming with special characteristic methods, such as dynamic movement primitive (DMP) (Schaal et al. 2005).

With a purpose to making robots complete operation tasks such as grabbing objects in dynamic and complicated environments, robots should have the ability of obstacle avoidance. The dynamic movement primitive (DMP) is used to model and generalize the motion trajectory inside the obstacle environment via combining the specific planning algorithm with its generalization and anti-jamming. In Kormushev et al. (2011), the DMP model combined with the synthetic capacity discipline method is used to comprehend the trajectory generation for the spherical impediment. In Stulp et al. (2013), the authors presented that the linear forcing term of the DMP can be represented by defining a function approximator. The method calls for unique obstacle records to calculate the repulsive pressure within the vicinity of the impediment. Gaussian mixture model (GMM) is broadly used in pattern recognition and facts evaluation, wherein, Gaussian mixture regression (GMR) is a widely-used quantitative analysing approach. The GMM can clean the probability distribution of arbitrary shapes. In Pervez and Lee (2018) and Hersch et al. (2006), authors developed a proposed Gaussian Mixture Model to represent the non-linear term, which is not required to manually specify the parameters of the basis functions. However, most probability estimation methods are often not able to attain whole information. For example, the sample is known, however the sample category of which Gaussian distribution is unknown. Hence it is desirable to employ the EM solution (most expectations value).

In addition, research in the field of human physical motions through tracking system has attracted public focus recently Yang et al. (2011). In Faion et al. et al. (2012), a research team developed a method, wherein a Bayesian based object tracking system with special focus on Microsoft KinectTM devices to intelligently schedule a network of multiple RGBD sensors. A kinematics based skeletal tracking system using Microsoft Kinect sensor with an upper limb virtual reality rehabilitation system has been investigated (Tao et al. 2013). In Raheja et al. (2011), a method of tracking the fingertips and palm centre has been developed using the Kinect sensor.

Dynamic Time Warping (DTW) is a measurement of the similarity between two time series. Its usage has been in the area of speech recognition to discover whether two words constitute the same phrase. In the time collection, the period of the two time series may not be identical, and the DTW calculates the similarity between the two time collection by using extending and shortening the time series (Calinon et al. 2007). In this paper, a Baxter robot has firstly been used to test the proposed teaching method by performing obstacle avoidance with different heights after the DMP is applied. Then a KUKA iiwa robot has been used to prove the achievement of our designed teaching method by drawing curves in a horizontal flat paper programming by recording a sequence of actions taught by a human demonstrator, after that we use the DTW and GMR to analyse and generalize the recorded movements. By doing this, the robot could playback in the vertical plan, which shows the success of our developed teaching interface.

## Preliminary

### Baxter robot

**Fig. 1:**
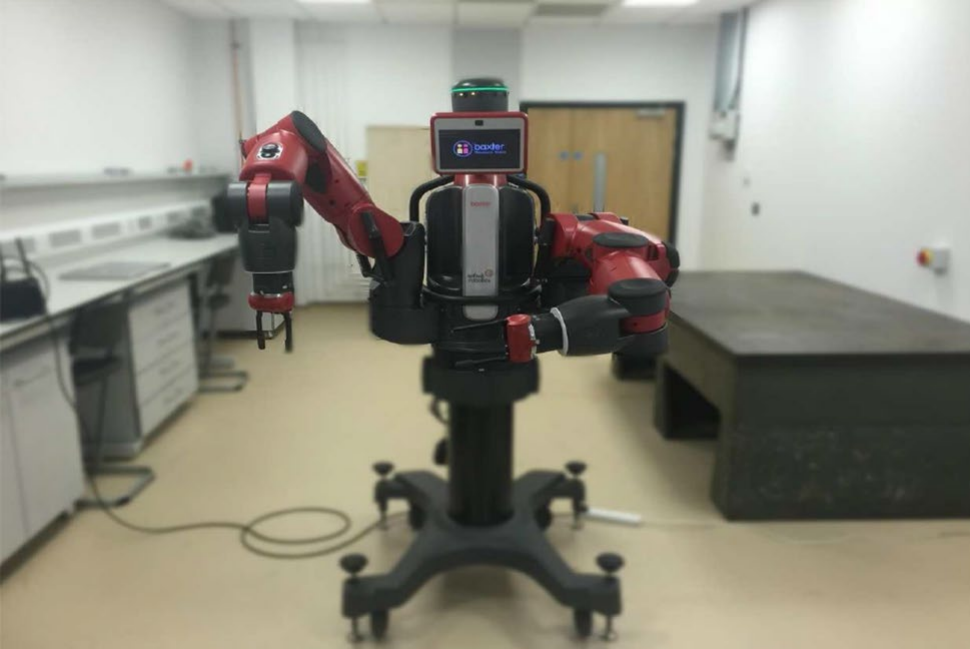
Image of Baxter robot

Baxter robot (Fig. 1) has been used mainly in the industrial research fields. The humanoid Baxter robot of Rethink Robotics is a popular collaborative robot (Li et al. 2017). The Baxter robot includes one torso, one 2-DOF head and two 7-DOF arms, which are shoulder joints: $s_{0}$, $s_{1}$, elbow joints: $e_{0}$, $e_{1}$ and wrist joints: $w_{0}$, $w_{1}$, $w_{2}$, respectively, running under the ROS and Linux operating systems (Li et al. 2017).

### KUKA iiwa robot

**Fig. 2:**
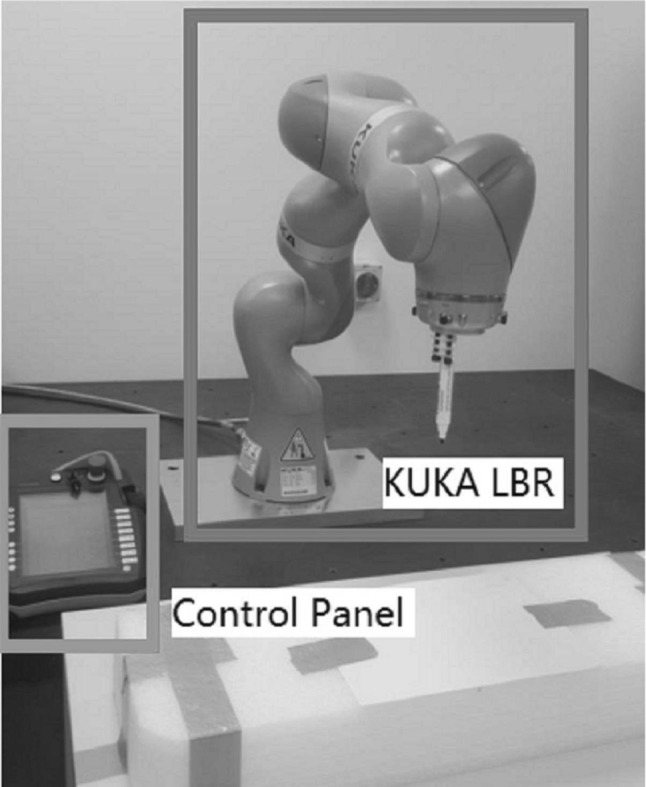
Image of KUKA iiwa robot

KUKA iiwa robot (with man-machine collaboration capabilities) is the first mass production of sensitive robots. The KUKA iiwa robot is able to achieve direct cooperation between human and robot to complete tasks of high sensitivity requirements (Schreiber et al. 2010). It has an advanced precision 7° of flexibility (DOFs) robot arm (Schreiber et al. 2010). The arm has been programmed via Workbench, which is a standard KUKA modifying platform employing KUKA robot language (KRL) and Java (Schreiber et al. 2010). The KUKA iiwa is controlled by the KUKA SmartPad shown in Fig. 2.

### Kinect v2

The Kinect v2 is an RGB-D device, released by Microsoft, which captures depth, colour, and IR images (Khoshelham and Elberink 2012). By use of the software development kit (SDK) released with the sensor, captured colour and depth information can be transformed into real-world frames, known as Camera Space. These frames are referenced to the centre of the depth sensor (Webb and Ashley 2012). The users also are able to obtain the skeletal tracking system via Kinect 2.0 SDK. This feature has been used in this paper, where the position of operators can be tracked by standing in front of the device.

### Gaussian mixture model

The Gaussian Mixture Model refers to the estimation of the probability density distribution of the samples, and the estimated model is the weighted sum of several Gaussian models (Ren et al. 2017). Each Gaussian model represents a class. The data in the sample are projected on several Gaussian models, respectively, and the probability of each class is obtained (Ren et al. 2017). Then the probability of the largest class for the results of the calculation can be selected by GMM Pervez and Lee (2018). In addition, the significance of GMM is to construct a series of GMMs to describe the joint density, and then obtain the probability density and regression function from each GMM.

The formulation of GMM is defined as,

1 $$\begin{aligned} p(x_{exp})=\sum ^{k}_{k=1}p_{k}p(x_{exp}\mid k) \end{aligned}$$

where *k* is the number of the model, $p_{k}$ is the weight of the $k_{th}$ Gaussian, and it is also the $k_{th}$ Gaussian probability density function, the average value of GMM is $\mu _{k}$, the variance is $\sigma _{k}$. Our estimation of this probability density is to require $p_{k}$, $\mu _{k}$ and $\sigma _{k}$ variables. When the expression is obtained, the result of the summation is the probability of the sample $x_{exp}$ belonging to each class.

### Dynamic time warping

The dynamic time regular method DTW is a typical optimization method. It describes the time correspondence between the test template and the reference template with the time regular function *W*(*n*) to solve the regular function corresponding to the minimum distance of the two templates (Petitjean et al. 2014). This algorithm is developed from dynamic programming (DP), and is widely used to match the templates with different lengths (Muda et al. 2010). It follows a classic algorithm for speech recognition.

**Fig. 3:**
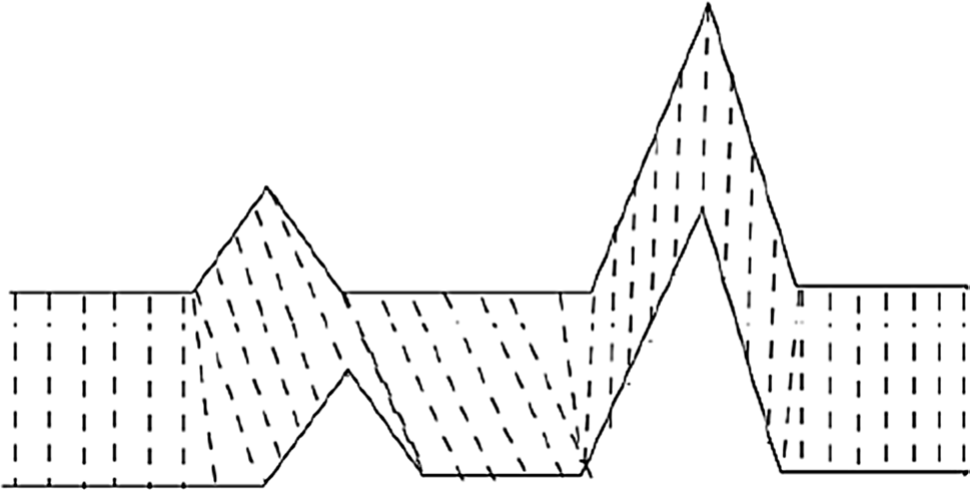
Warping example between two time series. Modified from Petitjean et al. (2014)

The DTW calculates the similarity between two time series by extending and shortening the time series. As shown in Fig. 3, the upper and lower solid lines represent two time series, and the dashed lines between the time series represent similar points between the two time series. The DTW uses the sum of the distances between all these similarities, which is called Warp Path Distance, to measure the similarity between the two time series.

### Dynamic movement primitive

DMP, that is a dynamical system developed from investigations into organic studies, learns from movement primitives to generate a sophisticated one (Mueen and Keogh 2016). The concept of movement dynamic primitives can be divided into two categories: constitute the states using unique formulations based on dynamical structures (Petitjean et al. 2014); and generating trajectories by way of interpolating the through factors (Ma et al. 2017). DMP consists of 2 components: a converted system *r* and a canonical system *h*. The formula is given as follows,

2 $$\begin{aligned} \dot{s}&=h(s) \end{aligned}$$

3 $$\begin{aligned} \dot{x}&=r(x,s,w) \end{aligned}$$

where *x* is the transformed system states, *s* is the canonical system states and *w* is the transforming parameters of the canonical system output.

## The enhanced teaching interface

In this section, we investigate the algorithm method for teaching process, playback and the generalizing task, including the DMP, the GMR and the DTW.

### Calculation of arm joint angles

**Fig. 4:**
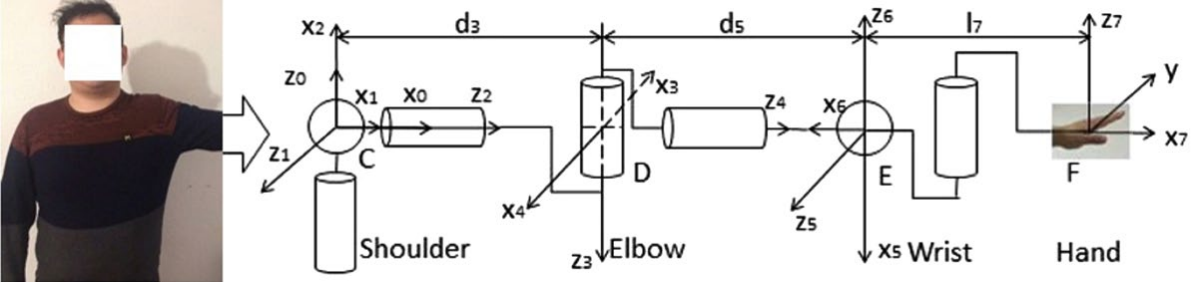
Human arm model and its DH coordinate frames modified from Li et al. 2016

According to our previous work in Li et al. (2016), a Denavit Hartenberg (DH) featured system chart has been created shown in Fig. 4 to represent the 7-DOF model of our human arm. The DH kinematic parameters of the human arm are indexed in Table 1. In step with the DH approach, the outline of the coordinate frames transformation from body *i* to border $i-1$ can be calculated.

**Table 1:** Model representation of the DH parameter table (Liang et al. 2016)

Link number	$\theta _{i}$	$d_{i}(m)$	$a_{i}(m)$	$\alpha _{i}(rad)$
1	$\theta _{1}$	0	0	$\pi /2$
2	$\theta _{2}$	0	0	$\pi /2$
3	$\theta _{3}$	$d_{3}$	0	$\pi /2$
4	$\theta _{4}$	0	0	$\pi /2$
5	$\theta _{5}$	$d_{5}$	0	$\pi /2$
6	$\theta _{6}$	0	0	$\pi /2$
7	$\theta _{7}$	0	$a_{7}$	0

**Fig. 5:**
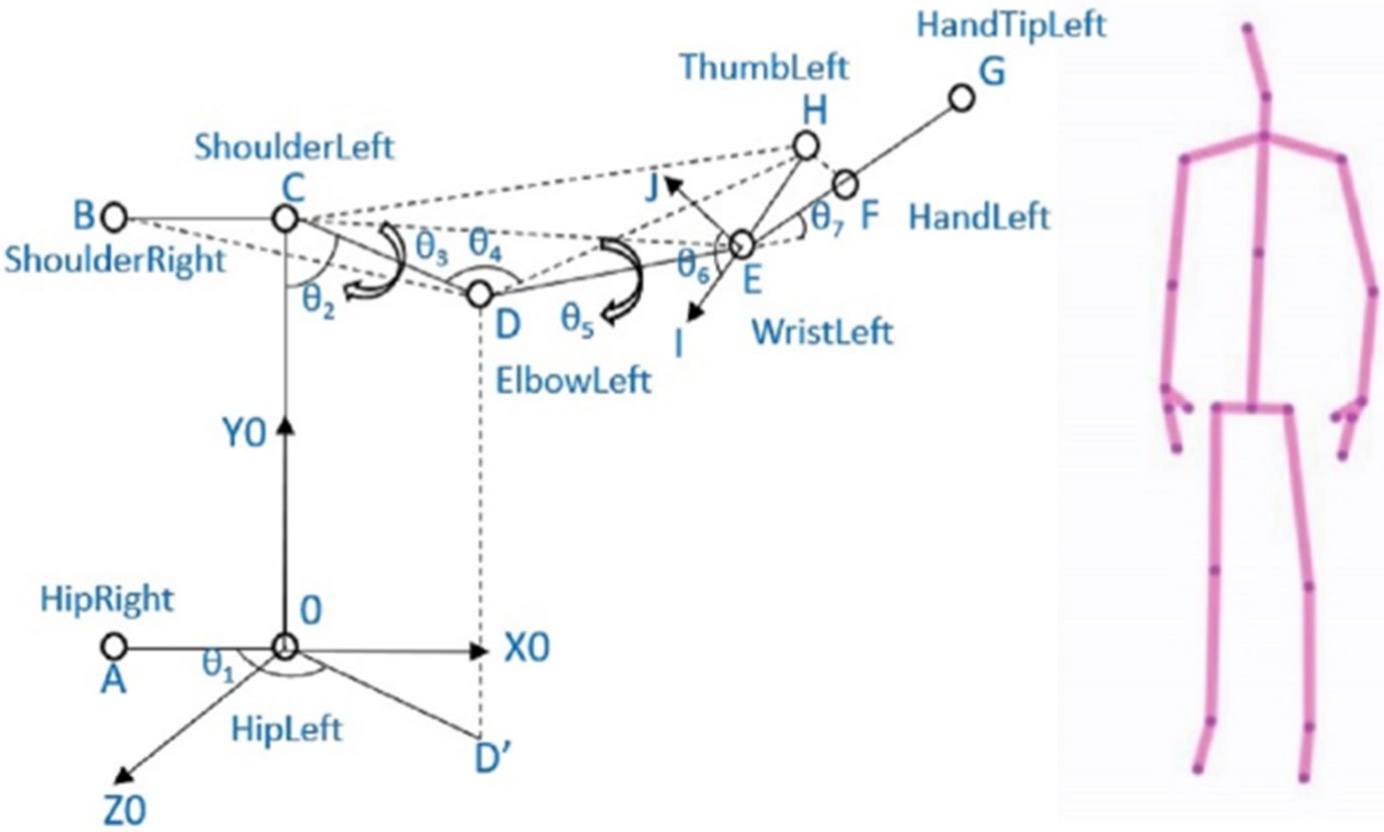
The geometry model for human arm in joint space and screenshot of skeleton tracking system modified from Li et al. 2016

The skeleton data of a human in 3-d positions could be obtained by using the Kinect sensor, which consist of 25 joints and this is shown in the right side of Fig. 5. Then, we make the arm model visible by creating the geometry model, which is shown in the left side of Fig. 5 in our previous work (Li et al. 2016). Next, the point Hip-Left is selected as the origin, at the same time as *x*-axis is within the identical path of vector $\overrightarrow{AO}$ and *y*-axis is in conjunction with vector $\overrightarrow{OC}$ (Li et al. 2016). It is simple to align the regular vector of every axis to the base coordinate, $\overrightarrow{X_{0}}$, $\overrightarrow{Y_{0}}$ and $\overrightarrow{Z_{0}}$ (Li et al. 2016):

4 $$\begin{aligned} \overrightarrow{X_{0}}=\frac{\overrightarrow{AO}}{\vert \overrightarrow{AO}\vert };\end{aligned}$$

5 $$\begin{aligned} \overrightarrow{Y_{0}}=\frac{\overrightarrow{OC}}{\vert \overrightarrow{OC}\vert };\end{aligned}$$

6 $$\begin{aligned} \overrightarrow{Z_{0}}=\overrightarrow{X_{0}}\times \overrightarrow{Y_{0}} \end{aligned}$$

In our previous work (Li et al. 2016), it was found that the plane COD and plane xOy form the supplement angle $\theta _{1}$.

7 $$\begin{aligned} \theta _{1}=\pi - <\overrightarrow{CO} \times \overrightarrow{CD}, \overrightarrow{CB} \times \overrightarrow{CO}> \end{aligned}$$

$\theta _{2}$ is the angle formed by vector $\overrightarrow{CD}$ and *y*-axis, which is shown as follows (Li et al. 2016):

8 $$\begin{aligned} \theta _{2}=<\overrightarrow{CO}, \overrightarrow{CD}> \end{aligned}$$

Similarly, the plane BCD and plane CDE form the angle $\theta _{3}$ in (Li et al. 2016).

9 $$\begin{aligned} \theta _{3}=<\overrightarrow{CB} \times \overrightarrow{CD}, \overrightarrow{CD} \times \overrightarrow{CE}> \end{aligned}$$

10 $$\begin{aligned} \theta _{4}=<\overrightarrow{DC}, \overrightarrow{DE}> \end{aligned}$$

$\theta _{5}$ is the angle between plane CDE and DEH Li et al. (2016).

11 $$\begin{aligned} \theta _{5}=<\overrightarrow{EC} \times \overrightarrow{ED}, \overrightarrow{ED} \times \overrightarrow{EH}> \end{aligned}$$

Angle $\theta _{6}$ is formed by vector $\overrightarrow{ED}$ and plane EFH according to Li et al. (2016).

12 $$\begin{aligned} \theta _{6}=\Pi /2 + <\overrightarrow{EH} \times \overrightarrow{EG}, \overrightarrow{ED}> \end{aligned}$$

However, the angle $\theta _{7}$ is difficult to calculate using the above method, which is the yaw angle of wrist (Li et al. 2016). $\theta _{7}$ could be treated as forming by $\overrightarrow{X_{5}}$ and $\overrightarrow{Y_{7}}$ (Li et al. 2016). While,

13 $$\begin{aligned} \overrightarrow{X_{7}}&=\frac{\overrightarrow{EF}}{\vert \overrightarrow{EF}\vert };\end{aligned}$$

14 $$\begin{aligned} \overrightarrow{Z_{7}}&=\frac{\overrightarrow{EF} \times \overrightarrow{EH}}{\vert \overrightarrow{EF} \times \overrightarrow{EH} \vert };\end{aligned}$$

15 $$\begin{aligned} \overrightarrow{Y_{7}}&=\overrightarrow{Z_{7}} \times \overrightarrow{X_{7}} \end{aligned}$$

so now the problem becomes to solve $\overrightarrow{X_{5}}$. We know that $\overrightarrow{X_{5}}$ is in the plane of EFH, and $\overrightarrow{X_{5}}$ is perpendicular to $\overrightarrow{DE}$. Supposing that:

16 $$\begin{aligned} \overrightarrow{X_{5}}= k_{1}\overrightarrow{EF} + k_{2}\overrightarrow{EH} \end{aligned}$$

There are:

17 $$\begin{aligned} (k_{1}\overrightarrow{EF} + k_{2}\overrightarrow{EH}) \times \overrightarrow{DE}=0; \vert k_{1}\overrightarrow{EF} + k_{2}\overrightarrow{EH}\vert =1 \end{aligned}$$

Therefore,

18 $$\begin{aligned} \theta _{7}=<\overrightarrow{x_{5}}, \overrightarrow{x_{7}}> \end{aligned}$$

By doing the calculation above, the every single joint angle is able to be obtained. Here we still need to consider two situations in order to make the calculations exact: (1) the left thumb must be inside the equal plane with the palm. (2) The angles of vectors change from zero to $\pi$, here further problems need be addressed (Li et al. 2016). Those above seven angles and their preliminary positions are shown in Fig. 4 in the joint space. The proposed geometry vector technique is based on the precept of cosine cost of two vectors proven in (11). Furthermore, the attitude among two planes may be calculated by giving their regular vector (Li et al. 2016).

### Pretreatment of the experimental data

The DTW algorithm is based totally on the concept of dynamic programming, and its purpose is to locate the shortest distance and highest quality matching path among two distinct check samples and reference templates. Let us define the reference time collection as $T=\left\{ t_{1},t_{2},t_{3},\cdots ,t_{i},\cdots ,t_{L_{1}} \right\}$ and the test sample as $R=\left\{ r_{1},r_{2},r_{3},\cdots ,r_{j},\cdots ,r_{L_{2}} \right\}$, wherein $t_{i}$ and $r_{j}$ denote the joint attitude values of the time factors, $L_{1}$ and $L_{2}$ denote the vector lengths. The space matrix *D*(*i*, *j*) collects when the vectors T and R are non-linearly matched is (Petitjean et al. 2014):

19 $$\begin{aligned} \begin{aligned} D(i,j)=d(t_{i},r_{j})+min\begin{Bmatrix} D(i,j-1)\\ D(i-1,j)\\ D(i-1,j-1) \end{Bmatrix},\\ i=1,2,\cdots ,L_{1};j=1,2,\cdots ,L_{2} \end{aligned} \end{aligned}$$

where $d(t_{i},r_{j})$ is the distance function of $t_{i}$ and $r_{j}$, the $D(L_{1},L_{2})$ is the shortest distance between T and R. The smaller the $D(L_{1},L_{2})$ is, the closer distance between T and R is.

In our research, the DTW approach has been used to align the recorded patterns by giving a warped characteristic $W=\left\{ w_{1},w_{2},\cdots ,w_{p},\cdots ,w_{P} \right\}$, where $w(p)= (i_{p},j_{p})$ is the match factor (Petitjean et al. 2014). Here the warped characteristic W is needed to decrease the gap between the check sample vector and the reference template vector, therefore the above equation may be rewritten as:

20 $$\begin{aligned} D=min\sum _{k=1}^{K}d[w(p)] \end{aligned}$$

where $d[w(p)]=d[T_{i}(p),R_{j}(p)]$ describes the distance measure between the $i(p)_{th}$ feature of the test sample vector and the $j(p)_{th}$ feature of the reference template vector, which is usually characterized by a square measure which is defined as follows:

21 $$\begin{aligned} d[w(p)]= [T_{i}(p)-R_{j}(p)]^{2} \end{aligned}$$

Assuming that the most desirable direction between the factors (1, 1) to (*i*, *j*) in the coordinate system is not related to the route after the point (*i*, *j*), the recursive method can be used to locate the most fantastic course. Here we outline the minimum cumulative distance among the two factors as $D_{Acc}(i, j)$, then we find that Petitjean et al. (2014):

22 $$\begin{aligned} D_{Acc}(i,j)=d(T_{i},R_{j})+min_{(q_{i},q_{j})}[D_{Acc}(q_{i},q_{j})] \end{aligned}$$

where $(q_{i}, q_{j})$ belongs to the set of all points within a certain path that exists between points (1, 1) and (*i*, *j*). It can be seen from the above components that the minimal cumulative distance of the factor (*i*, *j*) is related to not only the local distance $d(T_{i}, R_{j})$ of the eigenvalues $T_{i}$, $R_{j}$, but also the minimal cumulative distance earlier than this point in the coordinate system Petitjean et al. (2014).

Hence we conclude that $(i, j-1)$, $(i-1, j)$ and $(i-1, j)$ for any point $c(p) = (i, j)$ in the coordinate system can reach the preceding point of *c*(*p*), so the selection of the preceding factor only needs to align with the three above factors. According to the equation below, we can calculate the equal DTW distance among the check pattern vector and the reference template vector, which is shown as follows:

23 $$\begin{aligned} {D}'=D_{Acc}(L_{1},L_{2}). \end{aligned}$$

### Trajectory generation

The canonical system of DMP is an exponential differential equation given by:

24 $$\begin{aligned} \tau \dot{s}= -\alpha _{h}s \end{aligned}$$

where $\tau$ > 0 is the temporal scaling factor, $\alpha _{h}$ > 0 is the stable parameter and *s* is the phase value varied from 0 to 1.

The transformed system is made up of two contents (Bodiroža et al. 2013): a spring damping system and a nonlinear term, which is described as follows in Cartesian Space:

25 $$\begin{aligned} \tau \dot{v}&=k(g-x)-cv+(g-x_{0})sf(s) \end{aligned}$$

26 $$\begin{aligned} \tau \dot{x}&=v \end{aligned}$$

where $x \in R$ is the Cartesian position, $x_{0}$ is the start position, $v \in R$ is the velocity of the robot end-effector, *g* donates the target, *k* and *c* are the coefficients for spring and damping respectively. The transformation function *f* presents the complex nonlinear system, and it transforms the result of the canonical system, which is given by:

27 $$\begin{aligned} f(s)=\sum ^{N}_{i=1}p_{i}l_{i} \end{aligned}$$

where *N* is the number of GMM, $p_{i} \in R$ is the weight, *l* is the variable value of the normalized radial.

After selecting the start line $x_{0}$ and goal *g* of the canonical system *s* = 0, and integrating the canonical system, we are able to generate a motion by the usage of the weight parameter (Hogan and Sternad 2012). The significance of DMP here is to obtain the nonlinear transformation characteristic *f*(*s*) through skill transfer from the demonstrator. However, there is an issue in creating the transformed system through the usage of more than one verified path (Yin and Chen 2017). Therefore we applied the GMM to overcome the above problems.

The GMM is the estimation of the probability density distribution of the samples (Reynolds et al. 2000). The estimated version is the weighted sum of numerous Gaussian models and every Gaussian version represents a class (Reynolds et al. 2000). In this paper, the joint probability of the nonlinear system is the teaching data encoded through GMM, and the records is reconstructed via GMR to generalize the movement trajectory. For any degree of freedom, given *j* teaching data factors $\xi _{j}= \left\{ s_{j}, f_{j} \right\}$, *j* $\in R$, where $s_{j}$ and $f_{j}$ had been defined in DMP segment, *N* is the number of records points contained in a single training, every teaching data $\xi _{j}$ follows the subsequent probability distribution:

28 $$\begin{aligned} p(\xi _{j})=\sum ^{k}_{k=1}p(k)p(\xi _{j}\mid k) \end{aligned}$$

where *p*(*k*) is the prior probability, $p(\xi _{j}\mid k)$ is the conditional probability distribution, which follows the Gaussian distribution, and *k* is the number of Gaussian model distribution. Thus, the whole set of teaching data can be expressed by the Gaussian mixture model as follows (Yu et al. 2016):

29 $$\begin{aligned} \begin{aligned} p(k)=\pi _{k}\\ \end{aligned} \end{aligned}$$

30 $$\begin{aligned} \begin{aligned} p(\xi _{j}\mid k)=N(\xi _{j},\mu _{k},\sum \nolimits _{k})=\frac{1}{\sqrt{(2\pi )^{D}\left| \sum \nolimits _{k} \right| }}\\ *e^{-0.5(\xi _{j}-\mu _{k})^{T}\sum \nolimits _{k}^{-1}(\xi _{j}-\mu _{k})} \end{aligned} \end{aligned}$$

where *D* is the dimension of the GMM encoding the teaching data. Here we used BIC method to obtain the value of *k* (Burnham and Anderson 2004).

31 $$\begin{aligned} \begin{aligned} S_{BIC}=-L(\xi _{j})+\frac{n(k)}{2}lgN\\ L(\xi _{j})=\sum _{j=1}^{N}lg(p(\xi _{j}))\\ n(k)=k-1+k(D+\frac{1}{2D(D+1)}) \end{aligned} \end{aligned}$$

where $L(\xi _{j})$ measures the model’s characterization of data, n(k) is the number of free parameters of the model, which is a measure of the complexity of the model.

The parameters of GMM need to be determined, which are denoted as $\left\{ \pi _{k}, \mu _{k}, \sum _{k} \right\}$. That is the $k_{th}$ component of prior probability, expectations and variance, respectively. The EM algorithm is used to estimate the GMM parameters, which are obtained by giving the maximum similarity estimation of the parameters in the probability model, expectations and variance, respectively.

The teaching data $\xi _{j}$ is used as the query point, and the corresponding spatial value ${\xi }'_{f}$ is estimated using GMR. It is known that $p(\xi _{j}\mid k)$ satisfies the Gaussian distribution, $$\left( {\begin{array}{c}\xi _{f,k}\\ \xi _{s,k}\end{array}}\right) \sim N(\mu _{k},\sum _{k})$$, where $\mu _{k}$= $\left\{ \mu _{f,k},\mu _{s,k} \right\}$, $\sum _{k}$=$$ \begin{Bmatrix} \sum _{f,k}&\sum _{fs,k} \\ \sum _{sf,k}&\sum _{s,k} \end{Bmatrix}$$, and the conditional probability of $\xi _{f}$ and *k* satisfies the Gaussian distribution as given $\xi _{s}$, *k* (Yu et al. 2016).

Then we have

32 $$\begin{aligned} \xi _{f,k}\mid \xi _{s,k}&\sim N({\mu }'_{f,k},{\sum }'_{f,k}) \end{aligned}$$

33 $$\begin{aligned} {\mu }'_{f,k}&=\mu _{f,k}+ \sum \nolimits _{fs,k}\sum \nolimits _{s,k}^{-1}(\xi _{s,k}-\mu _{s,k}) \end{aligned}$$

34 $$\begin{aligned} {\sum }'_{f,k}&=\sum \nolimits _{f,k}-\sum \nolimits _{fs,k}\sum \nolimits _{f,k}^{-1}\sum \nolimits _{sf,k} \end{aligned}$$

where we could calculate the variance ${\sum }'_{f}$ and the average ${\mu }'_{f}$ of the *k*_*th*_ GMM component, which is shown as follows (Yu et al. 2016):

35 $$\begin{aligned} {\mu }'_{f}&=\sum _{k=1}^{K}\eta _{k}{\mu }'_{f,k} \end{aligned}$$

36 $$\begin{aligned} {\sum }'_{f}&=\sum _{k=1}^{K}\eta _{k}^{2}{\sum }'_{f,k} \end{aligned}$$

37 $$\begin{aligned} \eta _{k}&=\frac{p(\xi _{s}\mid k)}{\sum ^{K}_{k=1}p(\xi _{s}\mid i)} \end{aligned}$$

where ${\mu _{f}}'$ is the estimation acquired through the distribution of the expected conditions, and $\xi _{s}$ is similar to the reconstruction of area values (Yu et al. 2016). The generalized data of points are ${\xi _{f}}',\xi _{s}$, which are included in the teaching data and able to produce a smooth motion trajectory under the covariance constraint ${\sum _{f}}'$.

## Experimental studies

A Baxter robot and a KUKA iiwa robot are used in our experiments to verify the effectiveness of the proposed method. Baxter has an advanced precision 7° of flexibility (DOFs) robot arm. The arm could be programmed utilizing Ubuntu operaion system, which is a standard Baxter programming platform employing python language. KUKA iiwa robot has man-machine collaboration capabilities. It is able to achieve direct cooperation between human and robot to complete the task of high sensitivity requirements. As for the experimental platform, PC operation system is Windows 10. There is also Kinect SDK for Windows, Visual studio 2013 and OpenCV library. The KUKA robot needs to be programmed via Workbench, which is a common modifying platform combined with KUKA robot language (KRL) and Java. The experiments are conducted under laboratory conditions under adequate lighting.

### Obstacle avoidance experiment

**Fig. 6:**
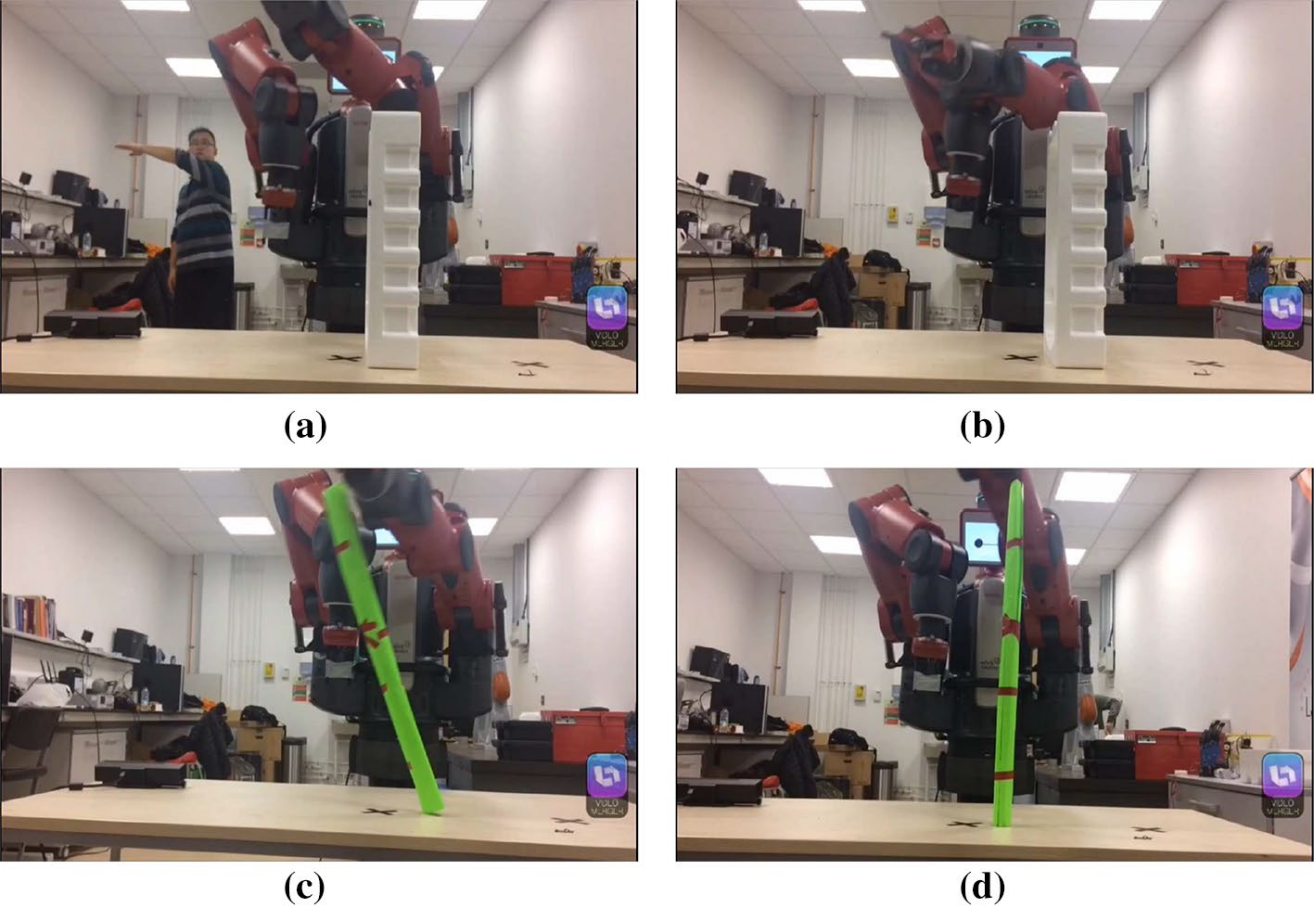
Illustration of the obstacle avoidance experiment.** a** Obstacle avoidance by teleoperation with Baxter.** b** Obstacle avoidance by playback.** c** Failed to pass the obstacle with increasing height. **d** Succeeded to pass the ob- stacle with increasing height after applying DMP

In this experiment, several tests have been designed to test the performance of our designed system by controlling the Baxter to avoid a high obstacle. Only one person stands forward to Kinect with a distance of about 2 m. Here, the operator guilds the Baxter to avoid the high obstacle by using teleoperation, which is shown in Fig. 6. At the same time, those data of each joints of operator’s arm is recorded, which is used for the playback of the Baxter robot. After that, we increase the height of the obstacle, it is noticed that the Baxter is not able to pass the obstacle successfully. Hence, the DMP has been employed to generalize the trajectory of the Baxter robot. By doing this, the Baxter is finally able to pass through the obstacle successfully with the increasing height.

### Trajectory generalizing experiment

**Fig. 7:**
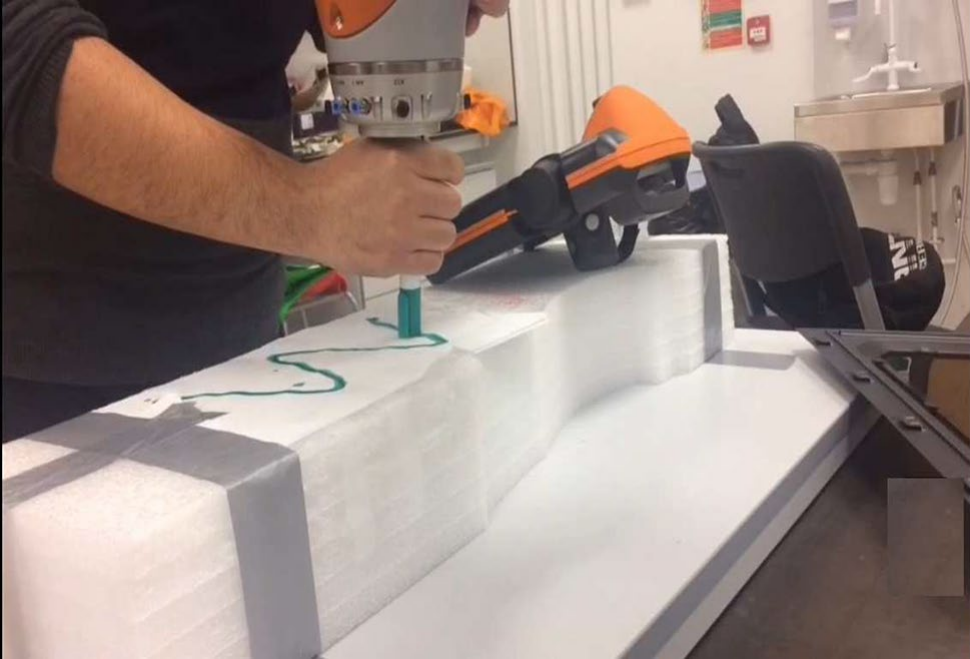
The setup of trajectory generalizing experiment

For this experiment, we test on a KUKA iiwa robot, the recorded data processed by using MATLAB from the movements of a demonstrator saved locally. Then the result data generated by the trajectories are sent to a separate computer and obtained access to control the robot arm. A marker pen is connected to draw patterns on a horizontal flat surface. A sine wave is chosen to be demonstrated, wherein the ability of the designed technique is tested extraordinarily with complex shapes. During the experimental process, we use a pre-revealed template shape on a sheet of A4 paper. Then, a human operator guides the KUKA robot to follow the template by handing the robot 5 times, which is shown in Fig. 7. The movement of the robotic endpoint is recorded at some stage in demonstration.

**Fig. 8:**
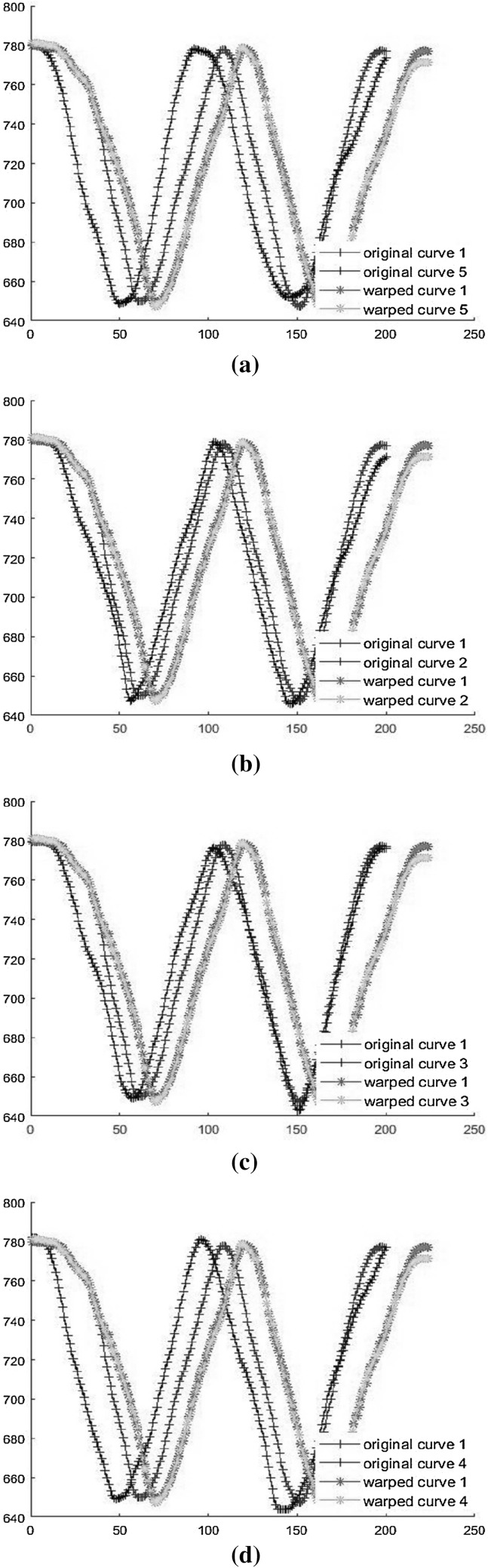
Illustration of the alignment using DTW.** a** Alignment result between the first and the second curves.** b** Alignment result between the first and the third curves.** c** Alignment result between the first and the forth curves.** d** Alignment result between the first and the fifth curves

The five recorded motion based trajectories in Cartesian space are saved, where the analysed data are initiated by K-means method and EM algorithm to obtain the GMMs. The experimental trajectories are plotted through MATLAB. After that, the DTW is used to align the 5 trajectories, where the first curve is selected as the reference to align all the other patterns, which is shown in Fig. 8 for the warping results. Then, GMM is used to encode the trajectories. Finally, the KUKA robot is able to reproduce a generalized curve on the vertical surface.

### Results

**Fig. 9:**
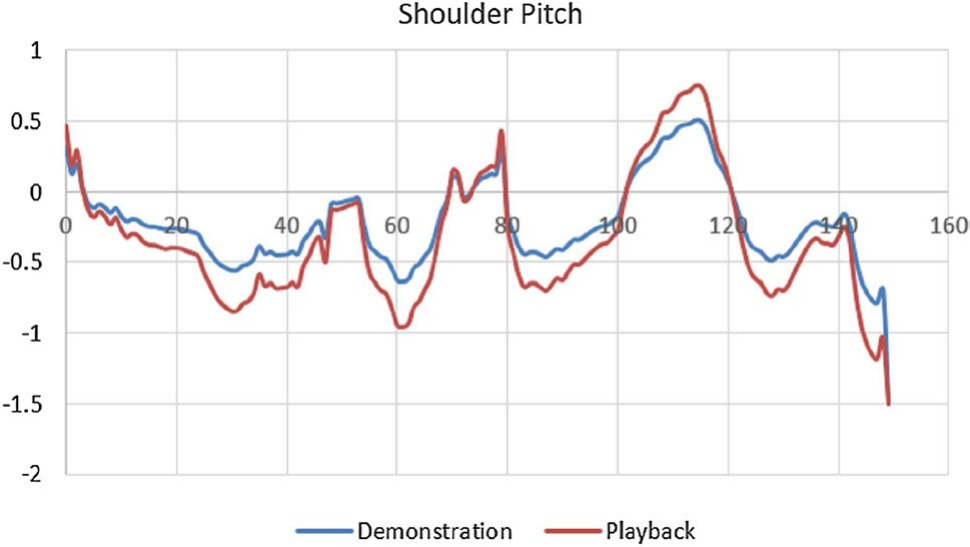
The learning and generalization result using the proposed DMP in an obstacle passing task

The first group of experiments aim to verify the performance of the proposed DMP, including the ability of generalization, i.e., spatial scaling, and the learning performance when the demonstration is defective. In this experiment, the demonstration process uses the joints *s*1, *e*0 and *w*2 fixed, and the angles of the joints *s*0, *e*1, *w*0 and *w*1 which are recorded during the process. Then the demonstration data are used for the training of the modified DMP. The training result is shown in Fig. 9. It can be seen from the graph that the maximum and the minimum values of Shoulder Pitch between the data from demonstration and playback, in some specific time point, are differing about 0.4 radians, meaning that the range of the arm motion of Baxter is increasing at about 0.4, which leads to an accurate motion. The motion of joint Shoulder Pitch is regenerated from the demonstration, which synthesizes the features of the demonstration and enables the robot to perform the obstacle passing task successfully as shown in Fig. 6d.

**Fig. 10:**
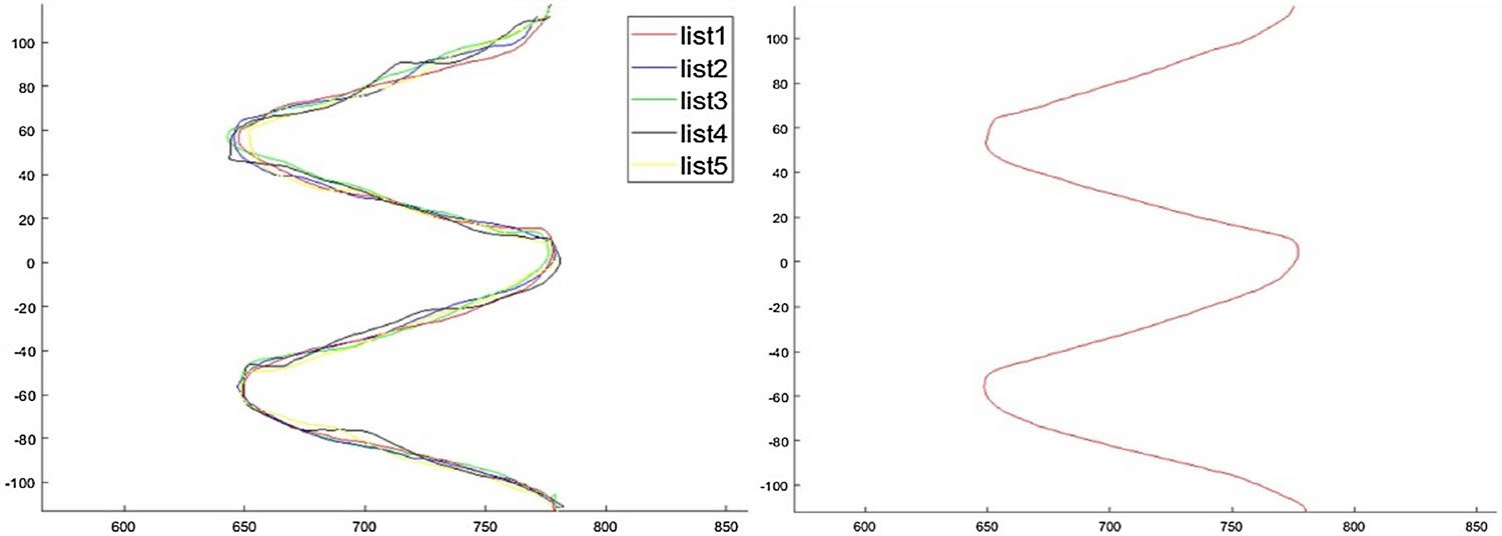
The demonstrated trajectories for the sine wave with GMM and the result

As shown in the left side of Fig. 10, for the second experiment, five distinctly separate curves are drawn horizontally. It can be concluded that, the number of GMMs effect the result trajectories. Hence, to achieve good performance of the generated trajectories, the number of GMM components is chosen as 20 in this paper. In addition, the optimal result trajectory is shown in the right side of Fig. 10. By using the GMR method, we transform the data retrieval problem of TbD into a joint distribution estimation problem, which is approximated by a mixture of Gaussians. During the calculation, the key point of learning process is correlative to the number of points in the sample set of data linearly. Here the prediction process relies on this number.

**Fig. 11:**
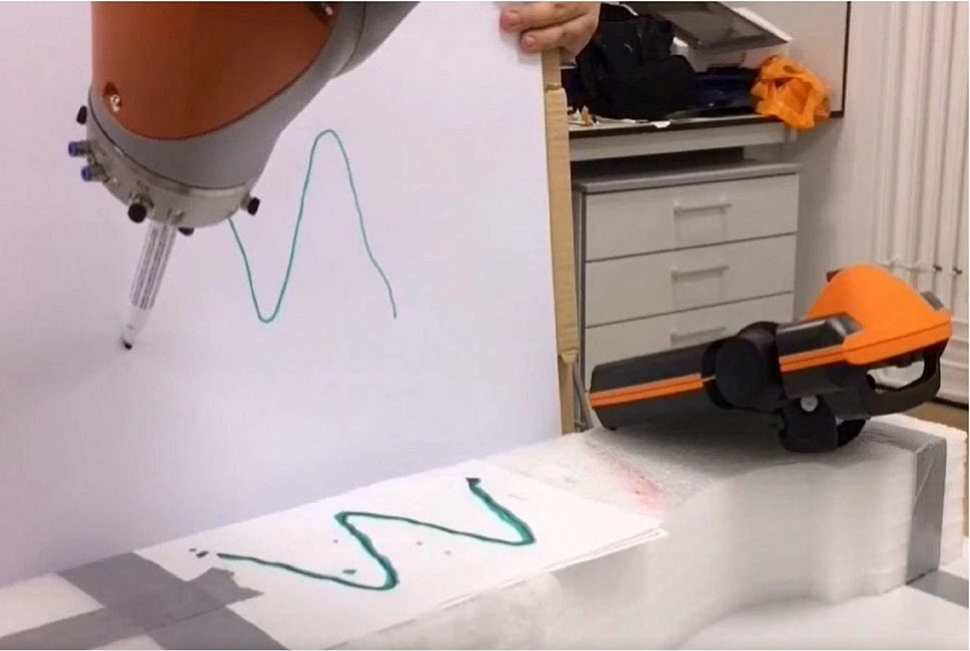
Curve on a vertical surface obtained after spatial generalization using the modified DMP

After that, we modified the DMP code to apply the spatial and temporal generalization. The generalized curve is then able to be drawn on a vertical flip chart pad by the playback process of KUKA robot, as is shown in Fig. 11, a smooth curve is retrieved from multiple demonstrations using the modified DMP, where the playback process can be achieved with 5 times’ speed, which is proved to achieve the proposed temporal generalization. In our future work, some teaching by demonstration based tasks would be researched by applying the DMP segmentation.

## Conclusion

A GMR and DMP combined with DTW based teaching by demonstration technology has been developed in this paper, which is an effective and superior method for humans to interact with the robot. The Kinect V2 sensor is used to teleoperate the Baxter so that the manipulator is able to achieve the generated motions more accurately. For the motion generation, the discrete DMP is selected as the basic motion model, which can achieve the generalization of the motions. To improve the learning performance of the DMP model, the GMM and GMR are employed for the estimation of the unknown function of the motion model. The DMP model is enabled to retrieve a better motion from multiple demonstrations of a specific task. Two experiments have been applied to test the performance of our designed teaching interface. The experimental results have verified the effective generalization of the proposed methods. Compared with the standard teaching approaches, the emphasis of the proposed teaching interface evaluates the DMP for multiple demonstrations by combining with GMR, meanwhile, all the experimental data have been initially pre-treated by DTW.
